# Skin gland concentrations adapted to different evolutionary pressures in the head and posterior regions of the caecilian *Siphonops annulatus*

**DOI:** 10.1038/s41598-018-22005-5

**Published:** 2018-02-23

**Authors:** Carlos Jared, Pedro Luiz Mailho-Fontana, Rafael Marques-Porto, Juliana Mozer Sciani, Daniel Carvalho Pimenta, Edmund D. Brodie, Marta Maria Antoniazzi

**Affiliations:** 1 Instituto Butantan, São Paulo, Brazil; 2 Utah State University, Logan, UT USA

## Abstract

Amphibian skin is rich in mucous glands and poison glands, secreting substances important for gas exchange and playing a fundamental role in chemical defense against predators and microorganisms. In the caecilian *Siphonops annulatus* (Mikan, 1920) we observed a concentration of enlarged mucous glands in the head region. In the posterior region of the body a similar concentration is made up of enlarged poison glands. These accumulations of glands structurally resemble the macroglands previously reported in anurans and salamanders. The skin glands in these regions are each surrounded by collagen walls forming a honeycomb-like structure. The collagen network in the head region firmly attaches to tiny pits in the bones of the skull. The two extremities of the body produce different secretions, containing exclusive molecules. Considering the fossorial lifestyle of caecilians, it seems evident that the secretions of the head and caudal region serve different functions. The anterior macrogland of mucous glands, rich in mucous/lipid secretion, in conjunction with the funnel-shaped head, may act to lubricate the body and penetrate the soil, thus facilitating locomotion underground. The blunt posterior end bearing an internalized macrogland of poison glands in the dermis may act in chemical defense and/or by blocking invasion of tunnels.

## Introduction

Caecilians are limbless amphibians comprising the Order Gymnophiona that are distributed in Southeast Asia, Central and South America and Africa^1–3^. They are fossorial animals, with compact skull, reduced visual system and a pair of sensory tentacles^2–5^. Possibly because of their fossorial habits and tropical distribution, caecilians constitute one of the least studied groups of vertebrates^6^. There are only 206 species (less than 3% of total extant amphibians) distributed in 10 families^7^.

As in other amphibians, caecilian skin is rich in glands which secrete substances that are fundamental to several vital functions, including chemical defense against predators and microorganisms^1,5,8,9^. Among amphibians, two basic types of cutaneous glands are present: mucous glands and poison glands. The mucous glands, in the form of typical acini, contain a characteristic lumen and secrete hydrophilic mucus that keeps the skin moist, facilitating gas exchanges^1,5,9,10^. The poison glands have no lumen and store their toxins in the form of granules (hence their traditional designation as granular glands)^9,11,12^.

Unlike the orders Anura and Urodela, Gymnophiona skin does not show any apparent glandular accumulations (macroglands), such as the parotoids, typically found in toads and salamanders and usually related to defense against predators^5,13–16^. It has long been known that granular glands are enlarged and most numerous in the posterior region of caecilians^17,18^. In this study we analyze the skin morphology and the biochemical composition of the cutaneous secretion of different regions of the body of the caecilian *Siphonops annulatus* (Fig. S1), a species widely distributed in South America^19^. Although this caecilian has a homogeneous body surface without protuberances, its head and posterior regions exhibit glandular accumulations in the dermis. In the head region the accumulation is comprised of mucous glands, whereas in the posterior region, it is comprised of poison glands. Biochemical differences also reflect specializations between secretions extracted from the two regions.

Taking into account the biology and natural history of *Siphonops annulatus*, we speculate about the role of glandular accumulations in this species and suggest that the composition of the cutaneous secretion in the head and posterior regions is related, respectively, to locomotion and defense against predators in the fossorial environment.

## Results

### Morphology

**Figure 1:**
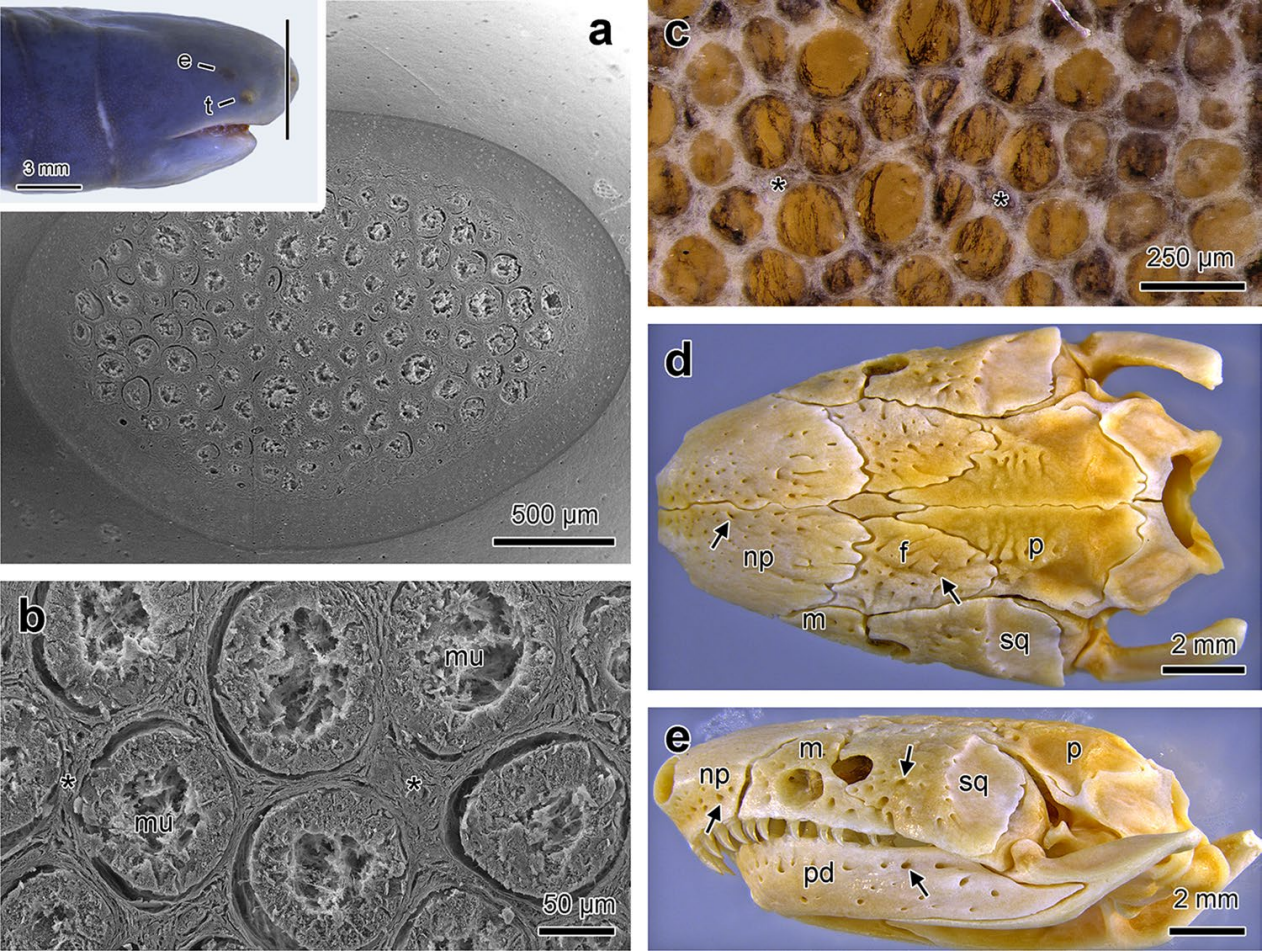
Internal morphology of the head skin and anatomy of the skull of *Siphonops annulatus*. (**a**) Internal aspect of the dermis of the snout tip revealed in transversal section, as exemplified in the insert. Note the large number of skin glands arranged side by side. (**b**) Higher magnification of (A) revealing the predominance of mucous glands (mu), which are identified by the presence of lumens. Note the collagen walls among the glands (*), conferring to the dermis a honeycomb appearance. (**c**) Image of a corresponding region of (**b**) after removal of the mucous glands, leaving only the collagen. (**d**–**e**) The skull of *S. annulatus* show many tiny orificies (arrows) mainly in the nasopremaxilla (np), maxillopalatine (m), frontal (f), squamosal (sq), parietal (p) and pseudodentary bones (pd). Eye (e), tentacle (t). Scanning electron microscopy (**a** and **b**), stereomicroscopy (a insert, **c**–**e**).

**Figure 2:**
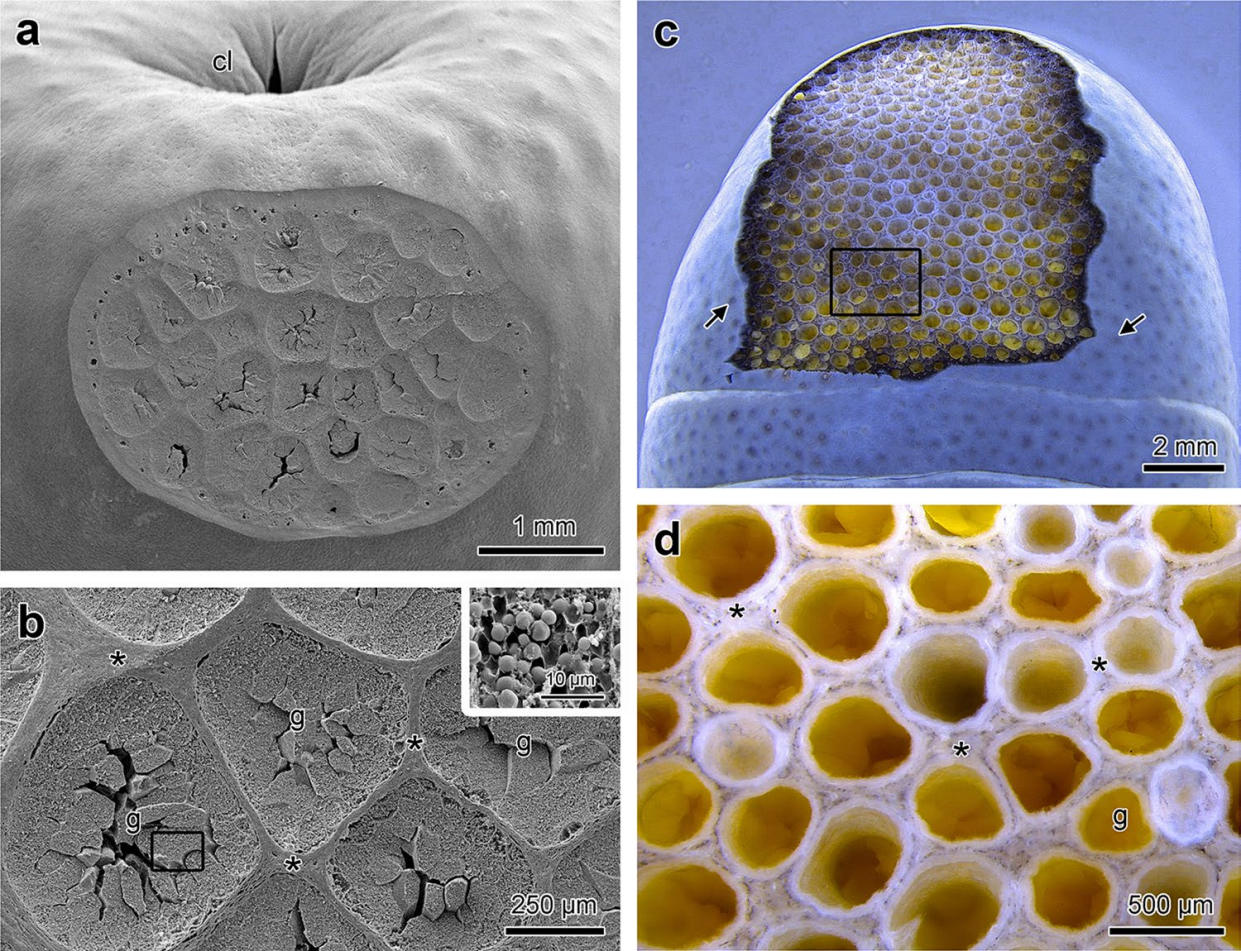
Anatomy of the skin of the posterior region of *Siphonops annulatus*. (**a**) Tangential section through the skin of the posterior tip of the body, just after the cloaca (cl), exposing a great number of large glands in the dermis. (**b**) Higher magnification of (**a**) revealing the poison glands (**g**), predominant in this region. Note the absence of lumens in the glands and the thick collagen walls (*) between glands. The insert shows a higher magnification of the poison stored in the glands in the form of granules. (**c**) The honeycomb architecture of the skin becomes more evident after removal of the poison from the glands. Note the presence of many pores in the skin surface (arrows), each one corresponding to a gland. (**d**) Higher magnification of the delimited region in (**c**) showing the honeycomb arrangement and the collagen walls (*), which remain unchanged after poison removal. Scanning electron microscopy (**a**−**b)**, stereomicroscopy (**c**−**d**).

The bluish-gray skin of *Siphonops annulatus* is smooth and shiny with well-developed annuli. In spite of the annuli, the surface of the body is homogeneous and, apart from the pair of tentacles, there are no visible protrusions. However, when the skin of the head and the posterior region is tangentially sectioned, a large number of densely packed glands are revealed within the dermis, forming honeycomb-like structures with distinctly different morphological characteristics at each end of the body (Figs 1 and 2). Each unit of the honeycomb is formed by collagen walls, surrounding each gland, either mucous or poison gland. At the head, the honeycomb structure exclusively contains large mucous glands identified by the presence of a characteristic central lumen, usually containing secretion (Fig. 1a,b). In contrast, at the posterior region, the honeycomb structure contains poison glands characterized by secretory cells completely filled with spherical granules and the absence of a lumen (Fig. 2a,b).

Both in the head and in posterior region, after gland removal, the dermal structure reveals the collagen walls composing the honeycomb/glands arrangement (Figs 1c and 2c). The collagen of the walls in the posterior region is much thicker (Fig. 2d) but more pliable than that of the head.

**Figure 3:**
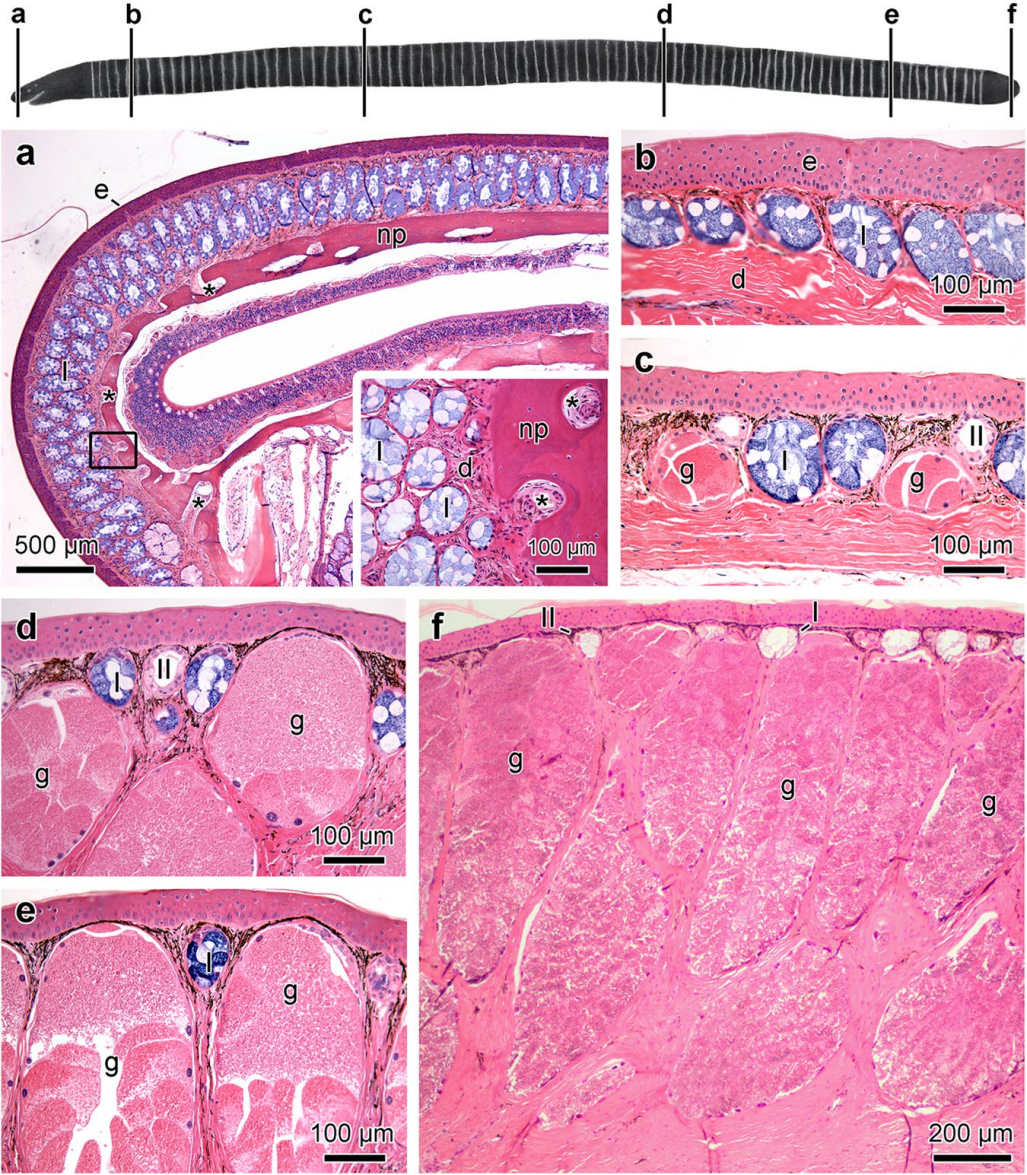
Characteristics and distribution of the cutaneous glands along the body of *Siphonops annulatus*. The letters (**a**−**f**) in the diagram, refer to the different regions of the skin analysed in the histological figures and correlate with the panels presented. (**a**) Sagittal section of the head. Note the predominance of Type I (**I**) mucous glands and the anchor spots (*) of the dermis (**d**) to the nasopremaxilla (np) bone. (**b**) Section of the skin, just posterior the head, showing abundance of Type I mucous glands (I). (**c**) Skin of the central-proximal region of the body, where the poison glands (**g**) and Type II mucous glands (II) are most frequent. (**d**−**f**) Moving posteriorly toward the body terminus, the poison glands (**g**) become progressively larger and more abundant. Epidermis (**e**). Stain: haematoxylin-eosin (**a**−**f**).

After total removal of the skin from the head, the skull of *S. annulatus* reveals that the bones form a single compact and robust structure (Fig. 1d) with a large number of tiny orifices, mainly distributed in the bones of the frontal, superior and lateral portions of the skull (Fig. 1d,e). The connective tissue matrix forming the honeycomb structure surrounding the glands extend into the pits of the skull anchoring the skin to the skull (Fig. 3a).

Microscopic evaluation of the skin reveals two types of mucous glands, Type I and Type II (Fig. S2), in addition to poison glands. Sections of the head reveal an accumulation of Type I mucous glands of much larger dimensions than those found in the rest of the body (Fig. 3a−f). Histological analysis of skin sections show that, in progression from anterior to posterior, Type I mucous glands progressively decrease in number and size (Fig. 3a–f), whereas poison glands become more numerous and larger (Fig. 3a–f). Specifically, toward the cloacal region, the poison glands are much enlarged (about 2.2 mm in height), occupying practically the entire volume of the dermis (Fig. 3f). Type II mucous glands are homogeneously distributed throughout the body, with the clear exception of the head (Fig. 3a–f).

The secretory epithelia of Type I and Type II mucous glands are composed of two cell types, which stain at different levels of affinity to PAS (Fig. S3A–B) and alcian blue (Fig. S3C). The two types of mucous glands stain poorly with bromophenol blue (Fig. S3D). In Type I glands, all cells of the secretory epithelia entirely stain with Sudan black, while in Type II glands only one kind of cell is strongly stained (Fig. S3E–B).

The morphological characteristics of poison glands, despite their variation in size, remain constant throughout the skin. They are always larger than the mucous glands and are also composed of two cell types (Figs S2A and S3D). One of these cell types is always located in an upper position in the gland, just below the duct (Fig. S3A), and has granules exclusively positive to PAS (Fig. S3B). The remainder of the glandular body is composed of cells full of granules that strongly react to bromophenol blue (Fig. S3D).

### Biochemistry

The cutaneous secretion extracted from the head is viscous, colorless, and transparent, while that extracted from the posterior region is more fluid, milky and opaque. The abundance of secretory proteins extracted from the posterior portion (11 mg/ml) is far greater than that from the head (0.2 mg/ml).

**Figure 4:**
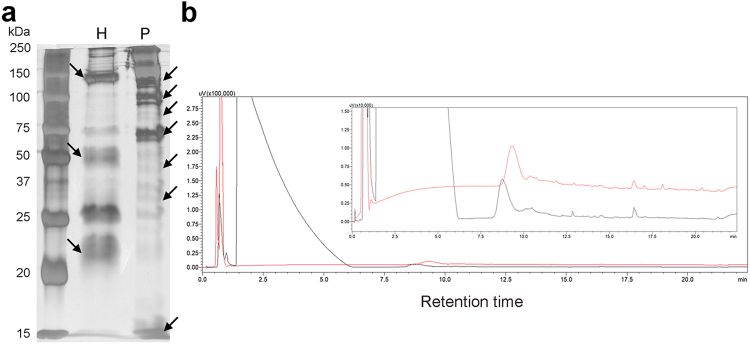
Biochemical characterization of the secretion extracted from the head and posterior region of *Siphonops annulatus*. (**a**) SDS-PAGE of the secretion of the head (H) and of the posterior region (P) (See Fig. 4S for the original image). The numbers on the left refer to the molecular mass markers (kDa) shown in the left column. Main differences between the two types of secretion are indicated by arrows. (**b**) C18-RP-HPLC profiles of secretions extracted from the head (red) and from the posterior region (black). The insert represents a high magnification of the image.

Electrophoretic profiles of secretions extracted from the head and from the posterior region show many differences in all regions of the gel, with several exclusive bands (Fig. 4a, Fig. 4S). Chromatographic profiles reveal significant differences between the head and posterior secretions, reinforcing their disparity. In the posterior region the secretion shows a majority peak in the polar region of the chromatogram (Fig. 4b). In addition, quantitative differences are found between the two secretions, which are most obvious at peaks eluted between 0 to 1 min and 8 to 22 min (Fig. 4b insert).

## Discussion

Among the amphibian orders, Gymnophiona is undoubtedly the least studied in all biological aspects. Skin morphology of caecilians remains practically unexplored despite marked and curious peculiarities compared to that of anurans and urodeles. Several of these peculiarities have been described in isolation but have never been analyzed in an integrative context. Some interesting caecilian skin features have been observed since the nineteenth century^20^. Following Sarasin and Sarasin^20^ and Ochoterena^21^, Sawaya^22,23^ observed heterogeneous distribution of glandular secretions and lethality in *Siphonops annulatus*. Sawaya^23^ experimentally demonstrated the poison of this species, both the mucous (extracted from the head) and the granular (extracted from the posterior region), is toxic to the caecilian itself, the toad (*Rhinella icterica*), the frog (*Leptodactylus ocellatus*) and the rat (*Rattus norvegicus*). Characteristics such as the heterogeneous distribution of poison and mucous glands along the body, and the glandular structure, have not received attention. In addition, fossoriality, a fundamental trait of caecilian biology has not been considered when analyzing glandular heterogeneity and distribution.

Amphibian granular glands in general produce a serous secretion that varies substantially in composition among species, though virtually always toxic^9^. Poison glands are present in all amphibian orders and have normally been associated with defense against predators^1,24^. In some anurans and salamanders such glands enlarge and accumulate in certain parts of the skin, forming macroglands, constituting prominent structures in relation to the body surface^9,12,25–27^. In *S. annulatus*, although the size of the poison glands varies throughout the body, the morphological and histochemical characteristics remain the same. Additionally, the morphology of the granular glands of *S. annulatus* is similar to that of salamanders and newts, showing a multicellular arrangement, different from that of anurans, which form syncytia.

Little is also known in relation to the chemical composition and activity of caecilian cutaneous secretions. Haemolytic activity was reported for *Siphonops paulensis*, probably as a function of a lysine-like protein^28–30^. Sawaya^23^ reported cardiotoxic activity in the cutaneous secretion of *Siphonops annulatus*, also demonstrating its paralyzing and lethal potential. In addition to these observations, it is known that the secretions of *S. annulatus* can cause strong irritation to the eyes and nasal mucosa (personal observations).

We verified the presence of proteins in the secretion of both body ends of *S. annulatus*, although proteins were more abundant in the secretion extracted from the posterior portion. Secretion extracted from the head (with a prevalence of mucous glands) and that from the posterior region (with prevalence of poison glands) are quite different, each one presenting exclusive proteins. Differences in composition of the two extremities were also evidenced by chromatography. The secretion of the posterior region is much more diverse and abundant when compared to the secretion extracted from the head. These data corroborate the observations of Sawaya^23^ who found greater lethality in the “milky” secretion from the skin of the tail.

*Siphonops annulatus* have the same diameter along the body (except for the smaller head), perfectly fitting the diameter of their tunnels, which allows their blunt posteriors to block the tunnel. Phragmosis, a defensive method consisting of blocking the entrance of a hole using part of the body^31^ has been reported for insects^31^, anurans (*Corythomantis greeningi*^32^, and even mammals such as the pink armadillo *Chlamydophorus truncatus*^33^. In the case of the tree frog *C. greeningi*, there is a strong relationship between the head, which is used in phragmosis, and the presence of poison gland accumulations^32,34^. Similar to the head of this tree frog, the posterior portion of *S. annulatus* has internalized glandular accumulations that are arranged in honeycomb architecture, resembling the typical parotoids of toads^15,27,35^. However, different from toads, protuberant glandular accumulations would not be evolutionarily favored in animals moving within tunnels such as caecilians.

The study of the structure and function of caecilian cutaneous glands has evolved since the works of A. Sawaya^22^ and P. Sawaya^23^ studying *Siphonops annulatus*. Sawaya^22^ showed that the posterior granular glands referred to as “giant glands” by Sarasin and Sarasin^20^) are found in the dorsal region of the last rings, close to the cloaca, decreasing in number towards the head. However, Sawaya^22^ did not suggest any possible reason for such glandular distribution.

Considering specifically the mucous glands, we found in *S. annulatus* two basic glandular varieties (Type I and Type II) which differ in size and composition of their secretions. This finding amplifies previous reports^22^ that observed only one type of mucous gland in this species. Here we demonstrate that Type I mucous glands produce lipid secretion in much greater amounts than Type II mucous glands. Type I glands are larger than Type II and accumulate in the head region, diminishing in size and frequency along the body. Mucous secretions, with the addition of lipid substances, may be efficient in reducing friction between the skin and the soil especially during the initial phase of burrowing, but also as the animal moves through the tunnels. In fact, we have observed in the field that the tunnels built by *S. annulatus* are always lined with a shiny and slippery secretion. Gabe^36^ studying *Ichthyophis glutinosus* skin morphology, suggested that the cutaneous secretion in general could be used in fossorial locomotion without describing any particular glandular distribution along the body.

In addition to the abundance of mucous glands, the head skin of *Siphonops annulatus* shows the presence of anchor spots of the dermis entering little pits in the skull bones. These anchor spots would maintain cohesion between the skin and the bones of the head during the constant friction between the head and the substrate when the animals move within the tunnels or when beginning excavation. Through the images showed by Wilkinson *et al*.^37^ it can be observed that such anchor spots are quite common in the skull of several other caecilians.

The morphological and biochemical aspects of the skin and cutaneous secretion of *Siphonops annulatus* presented in this work can be clearly related to the fossorial environment in which these animals live. While the funnel-form head mechanically opens the way through the soil, the lubricant mucous/lipid secretion produced by the mucous glands is spread over the body promoting efficient “diving” underground. In the posterior region, the granular glands form an internalized, non-protuberant macrogland, providing a defensive chemical mechanism against predators. At the same time, the poisonous body terminus can be used as a mechanical barrier, blocking the tunnel and preventing invasion by co-specifics or potential predators.

## Material and Methods

### Animals and extraction of cutaneous secretion

Eight adult *Siphonops annulatus* (375–452 mm, mean 410 ± SD 25 mm) were collected in Ilhéus (BA) and maintained in the animal house of the Laboratory of Cell Biology of Instituto Butantan. Crude skin secretions from all specimens were collected from both head and posterior regions of the body (secretions from each body region were pooled for all specimens). Each extremity was separately submerged in ultrapure water poured in a Petri dish, and the skin was stimulated to release secretion by gently brushing with a soft toothbrush. The resulting secretions were lyophilized and kept at −20 °C. One month after secretion collection, the animals were sacrificed using lethal doses of thiopental (30 mg/Kg) and preserved in 4% Bouin fixative or 10% buffered paraformaldeyhyde (pH 7.2) for 24 h. All procedures were approved by the Ethics Committee for the Use of Animals of Instituto Butantan (CEUAIB, 174/2004 e 444/2008) and all methods were performed in accordance with the relevant guidelines and regulations.

### Anatomical study

Following sacrifice, the head and the posterior portion of two individuals were separated from the body. In each extremity, tangential sections to the skin were made in order to expose deeper skin layers where the glands are present. Subsequently, the exposed area was cleaned with a toothbrush, removing the glandular content. In order to examine the skull, the head was cleaned of tissue by successive washings in sodium hypochlorite. Samples were analyzed and photographed with a Leica® M205-A stereomicroscope using the software LAS (Leica®).

The skin of the head and the posterior portion of two other preserved specimens was tangentially sectioned as described above. Heads and posterior regions were then dehydrated in a critical point dryer, sputter coated with gold, and examined under a scanning electron microscope FEI Quanta 250, operating at 10 kV.

### Histological study

After fixation, four samples of dorsal skin were removed along the body of four individuals. Head and posterior portions were removed and decalcified in 4% EDTA solution, pH 7.2, for two months. All samples (dorsal skin fragments and decalcified heads and posterior portions) were embedded in paraffin or historesin Leica®. Sections 2−5 μm thick were stained with haematoxylin-eosin and toluidine blue-fuchsin.

Histological sections were stained as follows: bromophenol blue for identification of proteins, periodic acid-Schiff (PAS) for identification of carbohydrates in general, alcian blue pH 2.5 for identification of acidic carbohydrates, and Sudan black for identification of lipids^38^. Slides were photographed with an Olympus BX51 microscope using Image Pro Express software.

### Electrophoresis (SDS-PAGE) and chromatography (RP-HPLC)

Lyophilized aliquots of secretion from the head and posterior region were dissolved in phosphate buffered saline and quantified by spectrophotometry using NanoVue Plus. Protein composition of skin secretions from the two areas was evaluated by electrophoresis in 12% polyacrylamide gel (PAGE) containing sodium dodecyl sulfate (SDS) under reducing conditions^39^. The gel was then stained with silver.

Crude secretions from head and the posterior regions were analyzed by reverse-phase liquid chromatography (RP-HPLC) using a binary HPLC system (20 A Prominence, Shimadzu Co., Japan). The sample was loaded on a C18 column (Phenomenex C18, 5 μm, 100 Å, 250 mm × 1 mm) and the content was eluted by a two-solvent system: (A) acetic acid (AA)/ H2O (1:999) and (B) AA/CAN/H_2_O (1:900:99) in a 0–90% gradient of solvent B over 20 min, after 5 min isocratic elution with 0% B. The flow rate was constant, set at 0.2 mL/min^−1^ at an oven temperature of 30 °C. The elutes were monitered by a Shimadzu SPD-M20A PDA detector scanning from 200 to 500 nm.

### Data Availability

The datasets generated during the current study are available from the corresponding author on reasonable request.

## Electronic supplementary material

Supplementary Information 
