# Supplementary material for: Skin gland concentrations adapted to different evolutionary pressures in the head and posterior regions of the caecilian Siphonops annulatus
Source: Sci Rep. 2018 Feb 23;8:3576. doi: 10.1038/s41598-018-22005-5 (PMC5824806; doi:10.1038/s41598-018-22005-5)
Supplement: Supplementary file 1 — Supplementary Information [file 41598_2018_22005_MOESM1_ESM.pdf]

SUPPLEMENTARY INFORMATION

Skin gland concentrations adapted to different evolutionary pressures in the head and posterior regions of the caecilian *Siphonops annulatus*

Carlos Jared[1], Pedro Luiz Mailho-Fontana[1], Rafael Marques-Porto[1], Juliana Mozer Sciani[1], Daniel Carvalho Pimenta[1], Edmund D. Brodie, Jr.[2*] & Marta Maria Antoniazzi[1]

[1] Instituto Butantan, São Paulo, Brazil

[2] Utah State University, Logan, UT USA

*Correspondence to: Edmund D. Brodie, Jr.

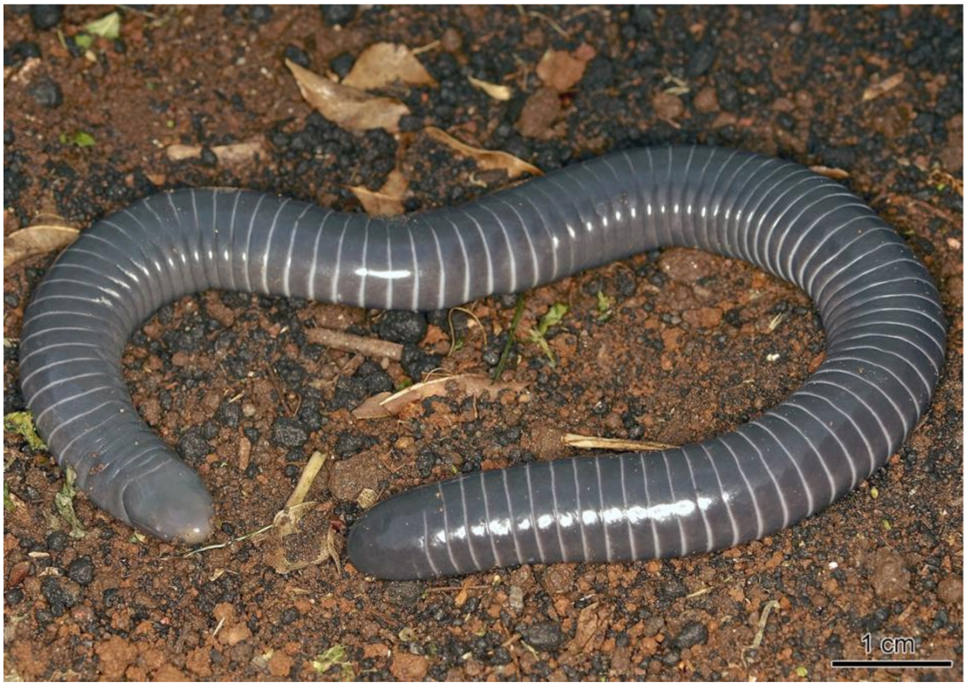

Figure S1.

Adult specimen of *Siphonops annulatus*.

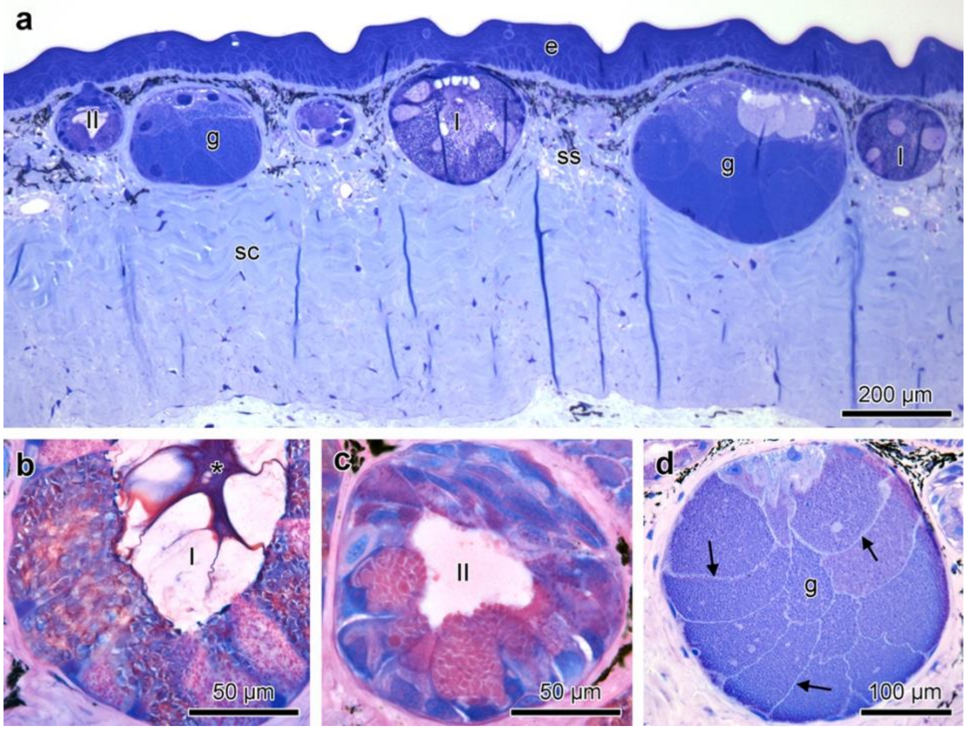

Figure S2.

Morphology of the skin of *Siphonops annulatus*. (a) Section of the skin showing the epidermis (e) and the spongy (ss) and compact (sc) strata of the dermis. In the spongy stratum three types of glands are observed: mucous glands of Type I (I) and Type II (II), and the poison glands (g). (b) Higher magnification of Type I mucous glands. Note the presence of secretion within the glandular lumen. (c) Higher magnification of Type II mucous glands. Note the smaller size and the absence of secretion in its lumen. (d) Morphological details of the poison glands. Note that the gland is composed of cells with clear delimitation (arrows), with absence of lumen. Stains: toluidine blue-fuchsin (a–d); haematoxylin-eosin (b–c).

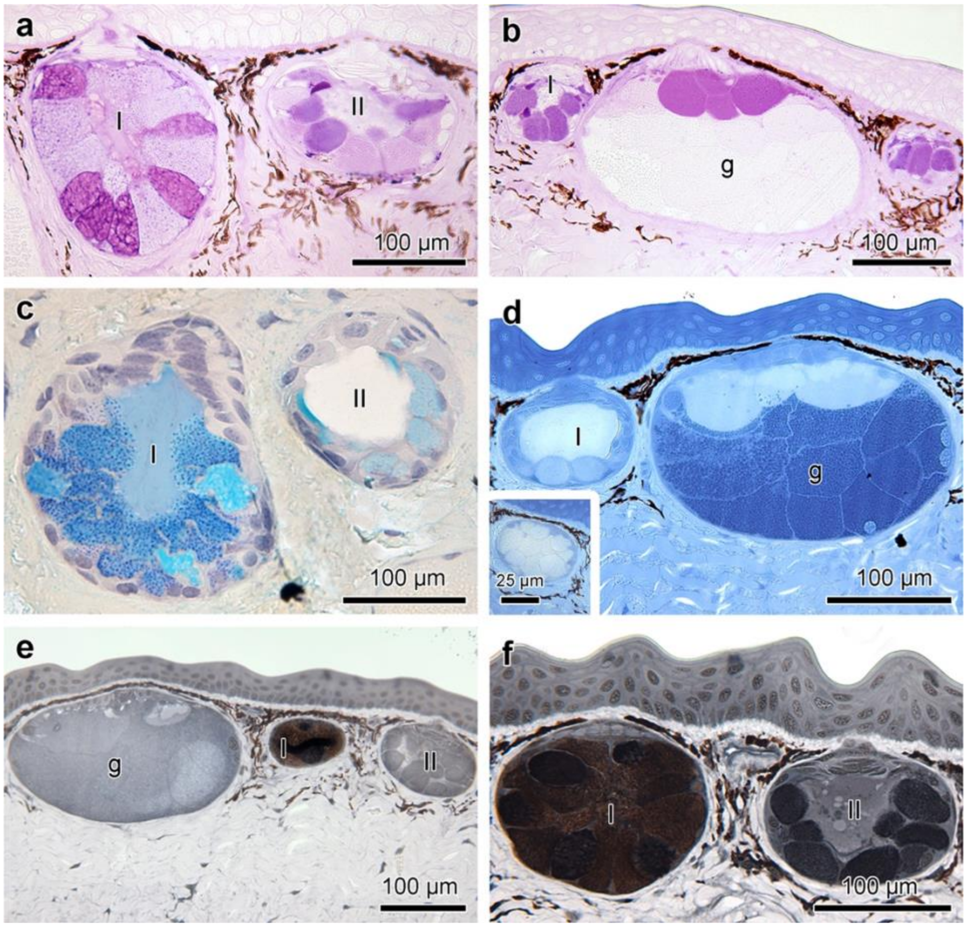

Figure S3.

Histochemical characterization of the cutaneous glands of *Siphonops annulatus*. (a and b) All cells of Type I (I) and Type II (II) mucous glands secrete neutral mucopolysaccharides. In the poison glands (g), only cells in the apical region produce mucopolysaccharides. (c) Both types of mucous glands (I and II) secrete acid mucopolysaccharides. (d) Most cells composing the poison glands indicate large amounts of proteins, except those in the apical region. (e and f) All cells of the Type I mucous glands exhibit lipid content, while in Type II, some cells are richer in lipids than others. Stains: PAS (a−b), alcian blue pH 2.5 (c), bromophenol blue (d), Sudan black (e−f).

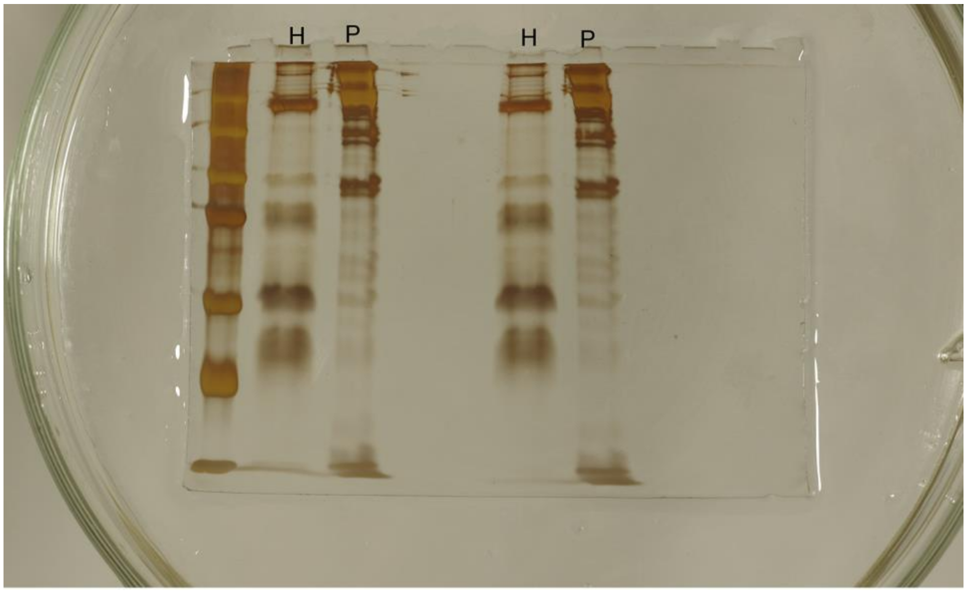

Figure 4S.

Original image of the gel used in Fig. 4. Head (H), Posterior Region (P).
